# Impact of Domain Size on the Length Scale of Local Glass Transition Perturbations Caused by an Immiscible Glassy–Rubbery Interface

**DOI:** 10.1021/acsmacrolett.5c00404

**Published:** 2025-08-18

**Authors:** Alexander A. Couturier, Connie B. Roth

**Affiliations:** Department of Physics, 1371 Emory University, Atlanta, Georgia 30322, United States

## Abstract

The depth-dependent profile in local glass transition temperature *T*_g_(*z*) was measured by pyrene fluorescence within 75 nm thick glassy polystyrene (PS) domains either capped by 600 nm thick poly­(*n*-butyl methacrylate) (PnBMA) layers or exposed to the free surface. In both systems, the total PS domain size is constrained by a “neutral” nonperturbing silica substrate. Remarkably, for this constrained PnBMA/PS bilayer system, we find the perturbing influence of the 6–7 nm PnBMA/PS interface to be essentially equivalent to that imposed by the free surface, in stark contrast to the previously reported long-range *T*_g_(*z*) perturbations of up to ≈250 nm for unconstrained glassy–rubbery interfaces between semi-infinite domains. For the 75 nm PS domains, both the PnBMA interface and free surface impart a local *T*_g_(*z*) reduction of ≈30 K, spanning ≈30 nm before bulk *T*_g_ is recovered, demonstrating that the total domain size strongly alters both the magnitude and extent of the dynamical gradient even when bounded by a nonperturbing interface.

## Graphical abstract

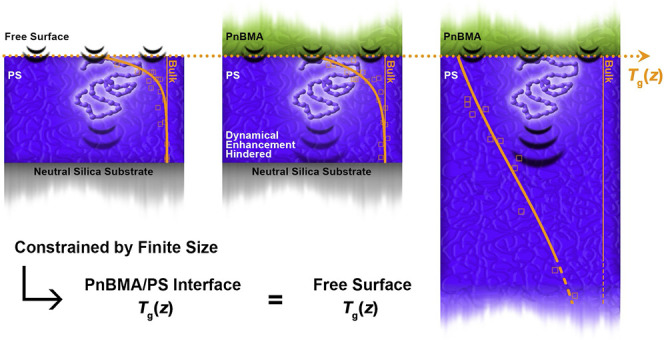

Understanding how perturbing interfaces alter local properties and dynamics is important for designing applications involving multicomponent materials with nanostructured morphology.^1–4^ In recent years, there have been considerable efforts made to determine how the local glass transition temperature *T*_g_(*z*) and dominant relaxation time τ­(*z*) vary with distance *z* from perturbing interfaces.^5–23^ It is thought that understanding the functional form of *T*_g_(*z*) and τ­(*z*) may provide fundamental insight into the glass transition and heterogeneous length scales that could be used to differentiate between different theoretical approaches.^5,7,16,17,24–27^ Recent work by McGuire et al. studying the film-average glass transition and physical aging of glassy–rubbery bilayers with finite layer thicknesses has suggested that the overall domain size of the layers may be an underappreciated variable explaining the large differences in *T*_g_(*z*) length scale for systems with glassy–rubbery polymer interfaces.^28^ In the present work, we experimentally map the local *T*_g_(*z*) profile as a function of depth *z* within 75 nm thick glassy polystyrene (PS) domains that are either capped by rubbery poly­(*n*-butyl methacrylate) (PnBMA) or simply exposed to the free surface. We demonstrate that by limiting the overall size of the domain, even with a nonperturbing “neutral” interface like a PS/silica substrate, the *T*_g_(*z*) gradient can be fundamentally altered.

Experimentally, there are limited techniques that can provide depth-dependent information on local dynamical gradients,^1,21,29–33^ where many experimental studies are instead forced to correlate average values measured for thin films with varying thickness to some anticipated depth-dependent functional form.^19,34–38^ One powerful method of obtaining a local glass transition temperature *T*_g_ involves the chemical labeling of polymer chains with pyrene dye, where *T*_g_ values determined by pyrene have been shown to be in good agreement with ellipsometry for thin films and differential scanning calorimetry (DSC) for bulk systems.^29,39–41^ The original measurements by Ellison and Torkelson demonstrated that the local *T*_g_ near the free surface of PS was reduced by ≈30 K and took a depth of ≈30 nm before bulk *T*_g_ was recovered.^29^ More recent measurements by Baglay and Roth using the same technique have shown that a glassy–rubbery polymer–polymer interface like PS next to PnBMA has a much stronger and longer ranged perturbation to the glass transition with the local *T*_g_(*z*) in PS being reduced by ≈60 K near the interface and taking until a depth of ≈250 nm before *T*_g_^bulk^ is recovered.^21,22^ In contrast, pyrene labeling of a glassy–rubbery block copolymer of poly­(methyl methacrylate) (PMMA) and PnBMA by Christie, Register, and Priestley has found that a ≈70 K *T*_g_(*z*) gradient can be squeezed into ≈13 nm lamellae domains.^42^

In this study, we also use pyrene fluorescence to measure the local *T*_g_(*z*) gradient as a function of depth *z* from the perturbing interface in 75 nm thick glassy PS domains. We juxtapose samples that are either capped by rubbery PnBMA or are simply exposed to the free surface. In both cases, the 75 nm PS domain is supported on silica, an interface that has been previously demonstrated to be “neutral” in that it does not perturb the local *T*_g_,^29,43–45^ thus providing a system with a single perturbing interface. The choice of domain size is influenced by the recent work of McGuire et al.^28^ that found the average *T*_g_ and physical aging rate of 75 nm PS layers capped with PnBMA to be equivalent to bulk, in strong contrast to the anticipated *T*_g_ reduction based on the *T*_g_(*z*) profile measured by Baglay and Roth across a rubbery–glassy PnBMA/PS interface between semi-infinite domains.^21^ We start in Figure 1 by comparing the local *T*_g_(*z* = 70 nm) values across three different types of samples. As illustrated by the schematics, samples were assembled to place the center of a 12 nm pyrene-labeled PS fluorescent probe layer at *z* = 70 nm from the perturbing interface within PS domains of total thickness of *h*_PS_ = 75 ± 2 nm. The PnBMA/PS bilayers of interest in this study are compared to PS films with a free surface by measuring the same sample prior to adding the PnBMA cap. These results are then compared to the analogous PnBMA/PS system with semi-infinite domains from the work by Baglay and Roth.^21^ We verified that the same *T*_g_(*z*) values are obtained for the PnBMA/PS bilayers even if the PS film is not measured first, and we also verified the results of Baglay and Roth.^21^

**Figure 1:**
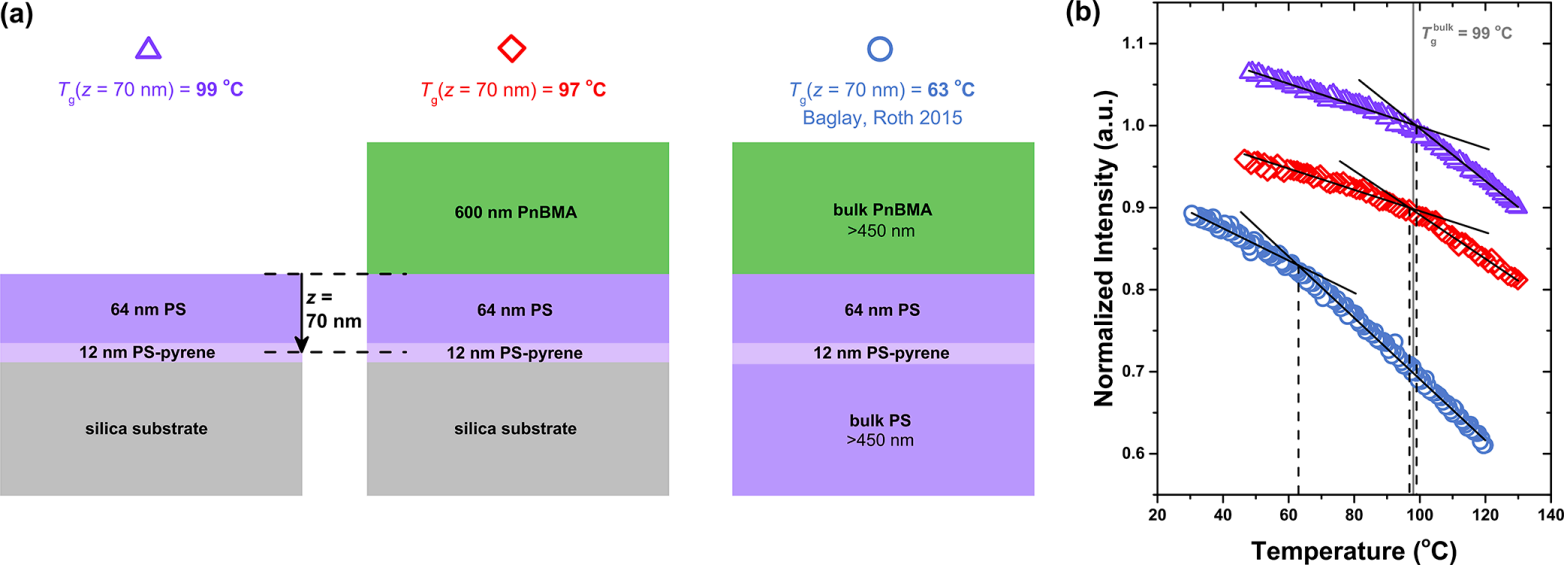
(a) Schematics of the different sample geometries assembled to place the midpoint of a 12 nm pyrene-labeled PS probe layer at a distance of *z* = 70 nm from the perturbing PnBMA/PS (red diamonds) or free surface (purple triangles) interface within 75 nm PS domains. The left and middle cartoons represent the same sample measured before and after the addition of a bulk PnBMA layer on top. These are compared to the Baglay and Roth^21^ samples (blue circles) with semi-infinite PS and PnBMA domains. (b) Normalized fluorescence intensity as a function of temperature collected on cooling at 1 °C/min (data shifted vertically for clarity) where the intersection of the linear fits to the glassy and rubbery regimes yields *T*_g_ for the fluorescent probe layer. Baglay and Roth data are reproduced from ref 21.

The multilayered samples were assembled by sequentially floating individually spin-coated layers of pyrene-labeled polystyrene (*M*_w_ = 672 kg/mol, *M*_w_/*M*_n_ = 1.3, 1.4 mol % pyrene^21–23^ ), neat PS (*M*_w_ = 1921 kg/mol, *M*_w_/*M*_n_ = 1.3), and 600 nm PnBMA (*M*_w_ = 319 kg/mol, *M*_w_/*M*_n_ = 2.6). Each PS layer was separately annealed under vacuum at 120 °C for ≈20 h prior to sample assembly, and PnBMA layers were similarly annealed at 65 °C. After the PnBMA capping layer had been added, the sample was annealed for 30 min at 120 °C to form the equilibrium interfacial width of 6–7 nm between the high molecular weight immiscible PS and PnBMA domains.^40,46^ These correspond to the same sample preparation and thermal history conditions used previously by Baglay and Roth resulting in the long-range *T*_g_(*z*) profile for the semi-infinite PnBMA/PS system.^21^ Previous work has shown that formation of the glassy–rubbery polymer interface is necessary to couple the dynamics of the two polymer domains and create long-range property gradients.^20,22,47–49^

To determine *T*_g_, fluorescence intensity was measured on cooling at 1 K/min by exciting pyrene dye with UV light at 332 nm and collecting at the wavelengths of 398 and 401 nm using 4 nm bandpasses. The fluorescence intensities plotted correspond to the sum of intensities measured at these two wavelengths, which is comparable to sampling the area under the fluorescence spectrum to the right of the second large peak around 395 nm. In this study, we have chosen to focus on this second peak because it is less sensitive to local polarity effects compared with the first peak,^21,50–52^ and we are collecting fluorescence intensity with the pyrene dye in close proximity to the polar PnBMA interface. We verified that this method produces the same film thickness dependent *T*_g_(*h*) results as previous data collection methods.^29,39,40^

Figure 1b plots the fluorescence intensity versus temperature for the three sample geometries shown. Following previous work, we identified *T*_g_ by determining the intersection of best fit lines to the glassy and rubbery regions of the data.^21–23,29,40^ The fitting windows for the fits were optimized by including as much data as possible within the glassy or rubbery region while minimizing the mean-squared error. We find that the PnBMA-capped *h*_PS_ = 75 nm sample yields *T*_g_(*z* = 70 nm) = 97 ± 3 °C, equivalent within experimental error to the same sample exposed to the free surface prior to adding the PnBMA layer with *T*_g_(*z* = 70 nm) = 99 ± 2 °C. Both of these values are equivalent to *T*_g_^bulk^ for PS of 99 ± 2 °C, defined from an average of bulk single-layer PS film measurements with thickness ≥150 nm. These results are consistent with previous work showing that a PS/silica interface does not perturb the local *T*_g_ of PS.^29,44,45^ However, interestingly, the PnBMA/PS bilayer result is at odds with expectations based on the *T*_g_(*z*) results by Baglay and Roth on semi-infinite PnBMA/PS domains that found the local *T*_g_(*z*) at *z* = 70 nm from the PnBMA/PS interface to be 63 ± 2 °C.^21^ If the silica interface is not perturbing *T*_g_, then this leaves the total PS domain size itself as the only obvious difference that could explain the stark difference in local *T*_g_ at *z* = 70 nm from the PnBMA/PS interface.

Figure 2 looks at the local *T*_g_ right next to the perturbing interface where the midpoint of the 12 nm thick pyrene-labeled probe layer is located at *z* = 6 nm. From the temperature dependence of the fluorescence intensity, we find the *T*_g_(*z* = 6 nm) = 74 ± 2 °C for the *h*_PS_ = 75 nm PnBMA/PS bilayer, comparable to the *T*_g_(*z* = 6 nm) = 77 ± 3 °C caused by the free surface. Again the *h*_PS_ = 75 nm PnBMA/PS bilayer result is strikingly different compared with the semi-infinite PnBMA/PS system where the local *T*_g_(*z*) evaluated at *z* = 6 nm from the PnBMA/PS interface was 38 °C.^21^ These results suggest that the strength of the perturbing interface is influenced by the total domain size of the PS layer.

**Figure 2:**
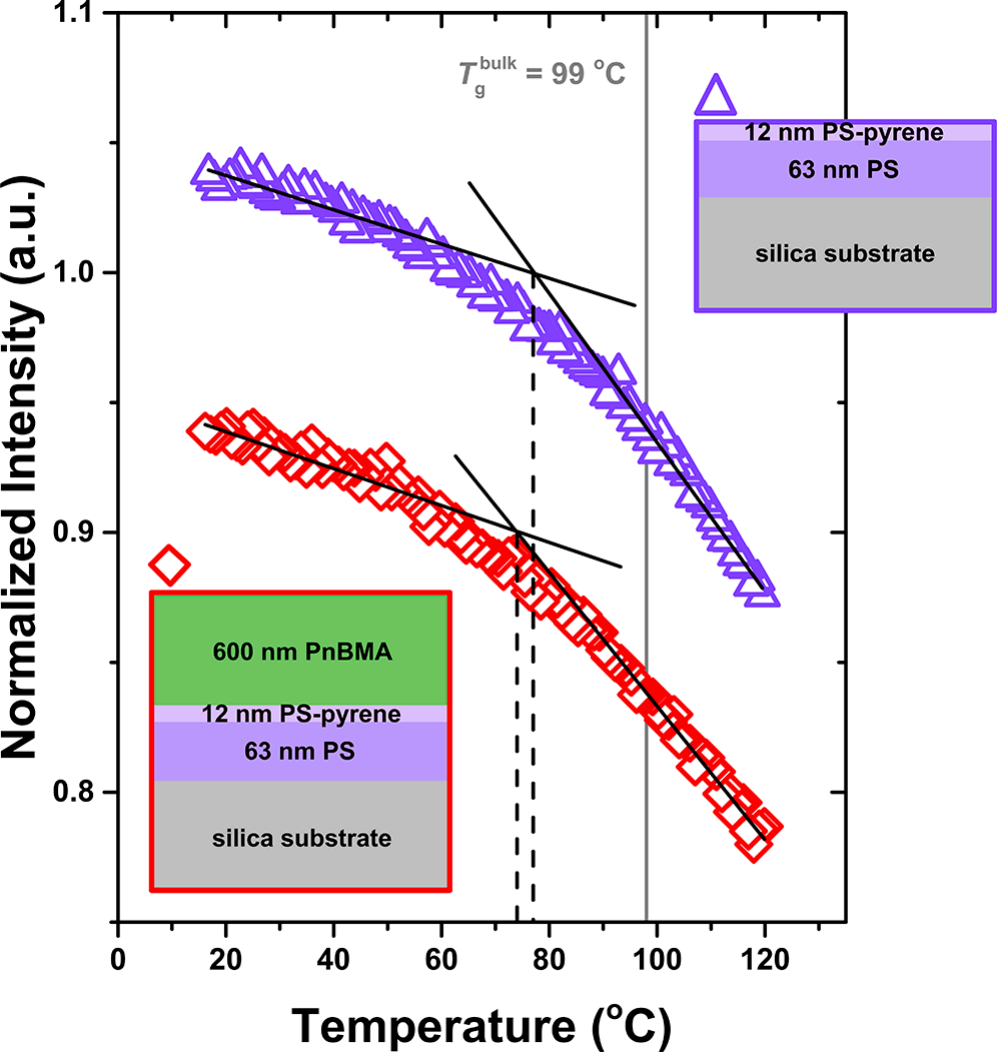
Pyrene fluorescence intensity versus temperature measured when the 12 nm pyrene-labeled PS probe layer is placed at the perturbing interface (midpoint of layer at *z* = 6 nm) within 75 nm PS domains yielding local *T*_g_(*z*) values of 74 ± 2 °C next to a PnBMA/PS interface (red diamonds) and 77 ± 3 °C next to a free surface (purple triangles). The PnBMA/PS interface is always annealed to equilibrium to form a 6–7 nm interfacial width.

To further understand how the *T*_g_(*z*) gradient is impacted by the finite size of the 75 nm PS domain, we varied the position of the pyrene-labeled probe layer to map the depth profile in local *T*_g_ across the PS domain. Figure 3 graphs *T*_g_(*z*) as a function of depth *z* from the perturbing interface, PnBMA/PS, or a free surface, within the 75 nm PS domains. The data are plotted with the *z* value of the symbols located at the midpoint of the pyrene-labeled probe layer and a horizontal error bar corresponding to the probe layer’s width. The vertical error bars represent the uncertainty estimates for each reported *T*_g_ value. In order to improve the resolution in *T*_g_(*z*), especially near the perturbing interface, some measurements were collected with a probe layer as thin as 6 nm instead of our commonly employed 12 nm probe layer. Surprisingly, the local *T*_g_(*z*) gradients for both the PnBMA/PS bilayer and PS film with *h*_PS_ = 75 nm are effectively identical within experimental error, recovering bulk *T*_g_ at *z* ≈ 30 nm. We find the measured *T*_g_(*z*) gradient can be well described by a saturating exponential function of the form

1 $$T_{g}\left(z\right)=T_{g}^{\mathrm{bulk}}-\Delta T_{g}^{\mathrm{int}}e^{-z/\xi_{\mathrm{int}}}$$

where *T*_g_^bulk^ is the bulk PS *T*_g_ value held fixed at 99 °C; Δ*T*_g_^int^ corresponds to the magnitude in *T*_g_ perturbation at the *z* = 0 interface; and ξ_int_ is the characteristic perturbation length scale of the interface. A saturating exponential functional form represents the most commonly reported profile for *T*_g_(*z*) from computer simulations^5,9,10,14,15^ and can be predicted by Schweizer’s Elastically Collective Nonlinear Langevin Equation (ECNLE) theory.^8^ However, we acknowledge that the experimental data could be adequately fit by other functional forms for *T*_g_(*z*). The best fit parameters for the PnBMA/PS bilayer and PS film geometries with *h*_PS_ = 75 nm domain size are comparable with Δ*T*_g_^int^ = 30 ± 5 °C and ξ_int_ = 13 ± 3 nm with the PnBMA cap and Δ*T*_g_^int^ = 30 ± 10 °C and ξ_int_ = 9 ± 2 nm for the free surface. It is worth noting that the dynamical gradient imposed by the free surface has been well-established to be independent of molecular weight.^20,39^ The similarity of the *T*_g_(*z*) gradients in the 75 nm PS domains suggests that the dynamical gradient imparted by the PnBMA/PS interface is likely not controlled by chain connectivity.

**Figure 3:**
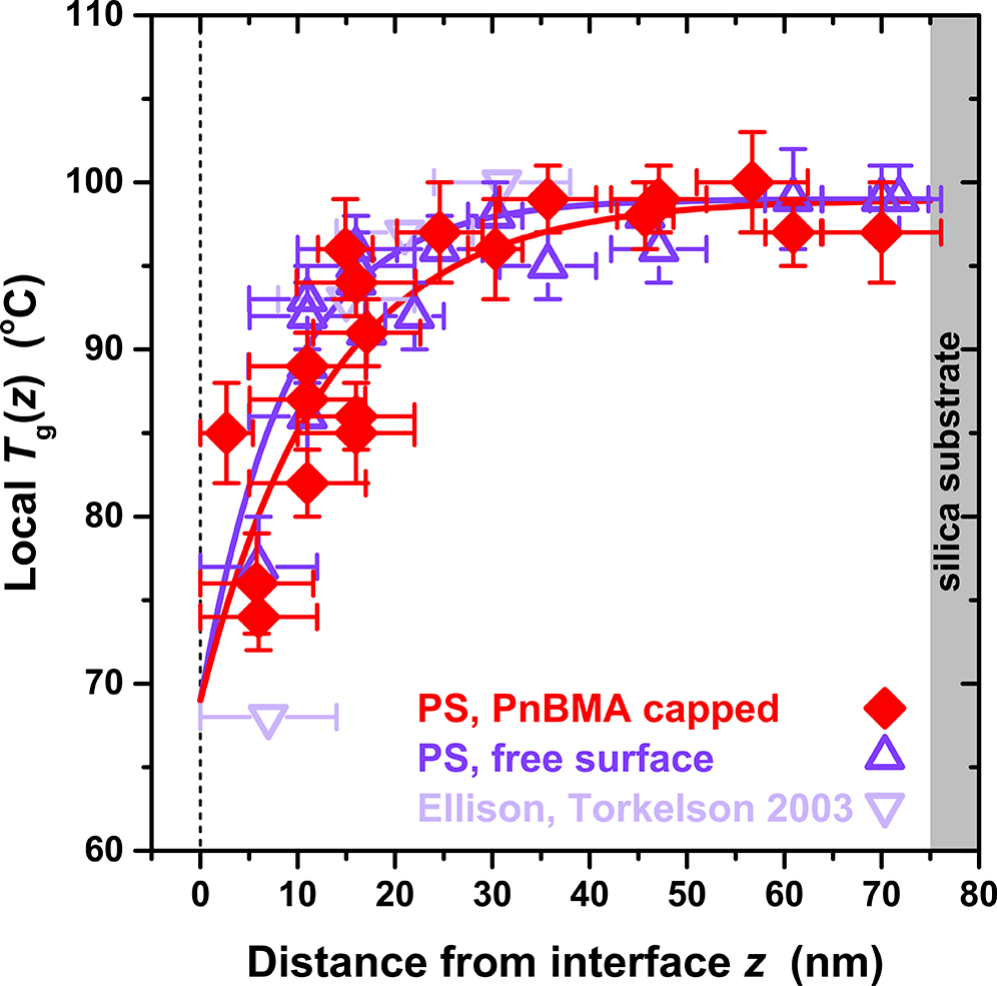
Experimentally measured gradient in local *T*_g_(*z*) within 75 nm PS domains either capped by 600 nm PnBMA (red diamonds) or exposed to the free surface (purple triangles). The position of the fluorescent probe layer was varied such that the total PS thickness was kept fixed at *h*_PS_ = 75 ± 2 nm, with the *z* value of the symbols corresponding to the midpoint of the pyrene-labeled probe layer where horizontal error bars depict the probe layer’s width. Four data points from the Ellison and Torkelson study^29^ are also included where 14 nm pyrene-labeled probe layers were placed near the free surface within bulk PS films >290 nm thick.

Included in Figure 3 are four data points from the Ellison and Torkelson study where the 14 nm pyrene-labeled layer was placed at different depths near the free surface of bulk films with thicknesses >290 nm.^20,29^ These data agree reasonably well with our measured *T*_g_(*z*) profile for a 75 nm thick PS film, suggesting the free surface *T*_g_(*z*) gradient in PS has already reached its maximum extent of ≈30 nm, and that 75 nm is not thin enough to significantly alter it. This conclusion is consistent with one of the other findings by Ellison and Torkelson that the free surface *T*_g_ perturbation is only altered in PS when the total film thickness is <60 nm.^29^ A similar finding was recently observed by Merrill et al. with ellipsometry where the glass transition breadth, thought to reflect the gradient in dynamics for films *h* < 60 nm thick, exhibited a narrower transition width in very thin films of *h* < 20 nm, consistent with a reduced dynamical gradient.^53^ In contrast, for a glassy–rubbery polymer interface, the range of the *T*_g_(*z*) profile extends up to ≈250 nm from the perturbing interface in semi-infinite systems where the domain size is >450 nm.^21,22^ In a separate study,^23^ Baglay and Roth also showed that the introduction of a second PnBMA/PS interface altered the *T*_g_(*z*) in PS at a distance of 100 nm from the interface when the PS domain size was <400 nm. We find now that this long-range *T*_g_(*z*) profile shrinks within a 75 nm PS domain that is too small to contain the full *T*_g_(*z*) gradient previously imposed by the PnBMA/PS interface. Figure 4 juxtaposes the *T*_g_(*z*) profiles from the *h*_PS_ = 75 nm PnBMA/PS bilayer with that from the semi-infinite system^21^ with *h*_PS_ > 450 nm, highlighting the vast contrast in *T*_g_(*z*) length scale obtained from the same glassy–rubbery interface depending on the total PS domain size. In addition, the magnitude of the local *T*_g_ perturbation at the interface is significantly different, reduced to only ≈30 K in the finite-size bilayer compared with more than 60 K for the semi-infinite system. Figure 4 includes data we collected at *z* = 134 nm on several samples with semi-infinite domain sizes confirming the long-range *T*_g_(*z*) profile previously reported.

**Figure 4:**
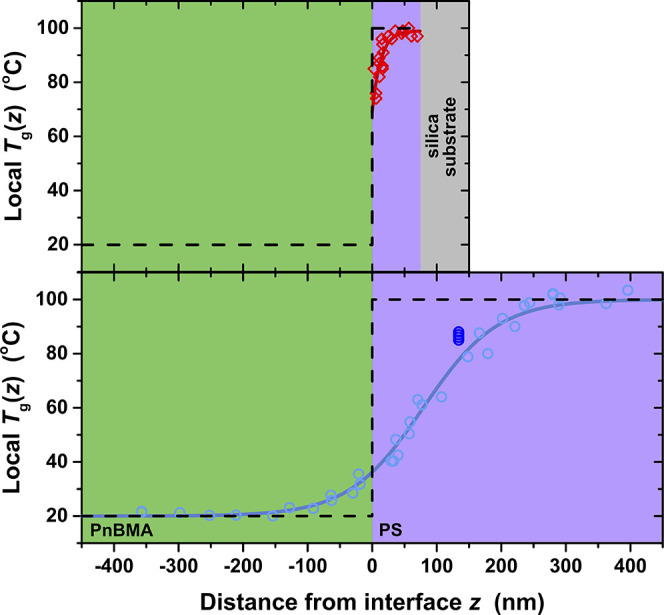
Measured *T*_g_(*z*) profile for the PnBMA/PS bilayer with *h*_PS_ = 75 nm finite domain size (red diamonds) including the fit to eq 1, compared to the fully extended *T*_g_(*z*) profile for the semi-infinite PnBMA/PS system with >450 nm domains (light blue circles) by Baglay and Roth^21^ including their hyperbolic tangent fit. Dark blue circles represent several data points we measured at *z* = 134 nm for the semi-infinite system to confirm the extended *T*_g_(*z*) gradient.

This observation of the unexpected constraint placed on the *T*_g_(*z*) profile by interrupting the PnBMA/PS interfacial perturbation with the silica substrate may help explain the physical aging results reported by McGuire et al. where a 150 nm PnBMA/75 nm PS finite-size bilayer exhibited bulk aging response.^28^ Integrating the *T*_g_(*z*) profile measured in Figure 3 for the *h*_PS_ = 75 nm PnBMA/PS bilayer gives an anticipated average $T_{g}\left(h_{\mathrm{PS}}\right)=\frac{1}{h_{\mathrm{PS}}}\int T_{g}\left(z\right)dz=94$ °C, close to *T*_g_^bulk^ and the measured average *T*_g_(*h*_PS_) = 97 ± 2 °C by McGuire et al. The possible impact that the PnBMA layer thickness also needed to be reduced for the McGuire et al.^28^ physical aging measurements is unknown at this time. This also does not address the question of whether film-average measurements of different properties might be biased toward slower or faster dynamics, as previously discussed.^28,36,54–56^ However, these new *T*_g_(*z*) results do demonstrate that the overall magnitude and extent of the dynamical gradient can be strongly impacted by the domain size, which has profound implications for making comparisons across different types of systems. For example, the range of this constrained *T*_g_(*z*) gradient within the PnBMA/PS bilayer with the *h*_PS_ = 75 nm domain agrees better with the majority of the literature investigating dynamical gradients than the long-range *T*_g_(*z*) profile previously measured in semi-infinite systems. Shorter range dynamical gradients reported in systems with small domain sizes^38^ such as block copolymer lamellae^42,57^ or computer simulations with periodic boundary conditions^12,18,58^ may likely reflect constrained *T*_g_(*z*) gradients where there is insufficient domain size to allow the full perturbation by the polymer–polymer interface to take effect.

The question remains, what is the underlying cause for the change in the dynamical gradient with overall domain size? Roth and co-workers have previously demonstrated that an interfacial width of ≈5 nm between glassy–rubbery polymer domains is necessary to get broad long-range material property gradients.^20,22,47,48^ For example, the *T*_g_(*z*) gradient was found to extend only 65–90 nm into the PS domain next to polydimethylsiloxane (PDMS) where the interfacial width was ≈1.5 nm, even for semi-infinite domain sizes.^49^ It has been shown that the finite thickness of layers within bilayer films can suppress the height of capillary waves at polymer–polymer interfaces, reducing the experimentally measured interfacial width.^59^ However, we estimate that the finite size of the 75 nm thick PS layer would not decrease the interfacial width of the PnBMA/PS interface by more than ∼10%,^59^ which we believe is insufficient to account for the large change in *T*_g_(*z*) gradient length scale. Alternatively, one possible explanation builds on the mechanism recently proposed by Gagnon et al.^47^ that acoustic waves near the boson peak,^60–62^ associated with collective vibrations believed to be precursors of α-relaxations,^62–67^ have wavelengths ∼5 nm that are able to propagate across a broadened polymer–polymer interface and couple the vibrational density of states (VDoS) between domains. In this picture, the breadth of dynamical gradients is in part controlled by the VDoS within the system, which is well-known to depend on the overall system size.^68^ In fact, both computer simulations and inelastic neutron scattering experiments have demonstrated that the VDoS near the boson peak changes in nanoscale systems depending on the type of confinement.^69–75^ The imposition of a rigid silica substrate to the system will cause acoustic waves to reflect at this sharp interface, altering the boundary conditions and VDoS of the system, thereby changing the nature of density fluctuations across the domain. Such a mechanism would be broadly applicable to the general nature of all glasses, consistent with recent work on small molecule stable glasses showing similar long-range perturbations to dynamics caused by a soft PDMS substrate.^76^ In contrast to studies that have tried to address the complexity of superimposing perturbations from two encroaching interfaces within finite domains,^6,8–10,17,23,77^ what is remarkable about this study is we demonstrate that even a nonperturbing “neutral” interface like the PS/silica substrate, which by itself does not perturb *T*_g_,^29,43–45^ can alter the *T*_g_(*z*) gradient in a fundamentally different manner by limiting the overall size of the domain and changing the nature of the boundary condition.
